# Examining Neuromuscular and Cortical Activity Over 1 Year Following Anterior Cruciate Ligament Reconstruction

**DOI:** 10.1111/sms.70358

**Published:** 2026-08-11

**Authors:** Aglaja Busch, Frank Mayer, Heiner Baur

**Affiliations:** ^1^ Sports Medicine & Sports Orthopeadics University of Potsdam, University Outpatient Clinic Potsdam Germany; ^2^ School of Health Professions, Division of Physiotherapy Bern University of Applied Sciences Bern Switzerland

**Keywords:** ACL, electroencephalography, electromyography, sensorimotor control

## Abstract

Cross‐sectional studies have indicated impaired neuromuscular and cortical activities following anterior cruciate ligament (ACL) reconstruction. However, adaptations throughout the recovery process remain unclear. Therefore, this study aimed to examine neuromuscular and cortical activity over the first year following ACL reconstruction. A prospective observational study was conducted, including 31 participants following ACL reconstruction (12 females, age: 25 ± 6 years, height: 173 ± 9 cm, mass: 72 ± 11 kg) and 31 healthy controls (12 females, age: 26 ± 6 years, height: 175 ± 9 cm, mass: 70 ± 10 kg). Electroencephalography and electromyography were used to assess cortical and neuromuscular activity during joint position sense (JPS) test, while only neuromuscular activity was recorded during stair descent and single‐leg hop tasks. The ACL group underwent evaluation at five postsurgical intervals (1.5, 3–4, 6, 9, and 12 months), while the control group was assessed on a single occasion. Linear mixed models were used to compute differences in neuromuscular and cortical activity across the various time points and groups in JPS testing and stair descent, while independent and dependent *t*‐tests were employed for analysis of the neuromuscular activity during single‐leg hop. ACL‐reconstructed participants demonstrated sustained altered neuromuscular activity in both involved and noninvolved limbs during JPS and stair descent tasks compared to controls (*p* < 0.05) with differences present across all measured time points. No significant differences in cortical EEG activity during JPS test were detected. Neuromuscular alterations were observed consistently throughout the initial year of recovery. The absence of cortical activity changes suggests a reassessment of more challenging tasks. Further investigation is required into neuromuscular and motor relearning protocols to ascertain their impact on rehabilitation and return to sport.

## 1 Introduction

A rupture of the anterior cruciate ligament (ACL) is a significant knee injury in sports [1]. As demonstrated in various studies, the incidence rate has been described as ranging between 32 and 91 per 100 000 people in different countries [2, 3, 4, 5]. Furthermore, a systematic review reported annual ACL injury incidences in amateur athletes to range between 0.03% and 1.62% [6]. This injury typically results in immediate symptoms such as knee joint swelling, restricted movement, and pain [7]. Despite extensive treatment that may include both surgical and nonsurgical approaches, reduced physical activity and lower knee‐related quality of life are often observed [8, 9]. Moreover, the risk of developing knee osteoarthritis is significantly higher following an initial ACL injury [10], and there is an increased likelihood of secondary injuries, as noted in the literature [11].

Moreover, authors have identified altered kinematics, kinetics and neuromuscular control during movement execution following ACL rupture and reconstruction [12, 13, 14]. Functional impairments such as reduced dynamic knee stability, altered landing mechanics, and compensatory movement strategies during gait and other daily activities have been identified [15, 16]. Such deficits are consistently observed across both low‐demand tasks, such as walking, and high‐demand movements, including stair walking or jump landing [12, 17, 18]. Moreover, alterations in neuromuscular control have primarily been observed in key knee‐stabilizing muscle groups, including the quadriceps (e.g., vastus medialis and vastus lateralis) and hamstrings (e.g., biceps femoris and semitendinosus) [14, 19], which play a central role in dynamic joint stability [7]. A systematic review reported reduced voluntary quadriceps activation shortly after reconstruction and continues to be diminished over time, with limited to moderate evidence supporting these observations [14]. In contrast, no clear consensus has been reached on the direction of quadriceps neuromuscular activity during tasks such as jump landing, stair ambulation, and walking [20, 21, 22, 23, 24]. With regard to hamstring neuromuscular activity and coactivation, increases with moderate to large effect sizes have been reported in ACL patients across various functional activities, including gait and stair ambulation [19]. Consequently, functional tasks such as walking, stair ambulation, and jump landing are commonly used to assess these impairments, as they impose varying mechanical and neuromuscular demands on knee joint stability.

The persistent knee dysfunction and the higher rates of subsequent injury are thought to be related to changes in somatosensory perception, adoptions within the central nervous system, and altered motor responses [25, 26]. Some evidence suggests changes in cortical electrical activity as well as brain activation patterns following ACL rupture [26, 27, 28, 29, 30, 31], highlighting the fact that it should not be considered a purely musculoskeletal injury [32]. In particular, increased activation in the primary motor cortex along with brain regions responsible for sensory‐visual–spatial processing has been identified following ACL rupture and reconstruction [30, 31, 32]. In addition, a greater degree of attentional control and enhanced processing of movement‐related and sensory information was observed, as evident by an increased theta‐power recorded in electrodes over the frontal cortical region of interest and a decreased alpha‐2 power in parietal cortical regions of interest in ACL reconstructed participants in comparison to a healthy control group during a joint position sense (JPS) task [27].

However, research on neuromuscular and cortical activity has typically been limited to periods after the return to activity or sport. There is a notable gap in knowledge regarding the progression of neuromuscular control and brain function throughout the rehabilitation process [33]. Furthermore, small sample sizes in cross‐sectional studies impede the ability to draw meaningful conclusions and comprehensively understand changes throughout the recovery process [34]. A comprehensive understanding of the evolution of neuromuscular and cortical function during the rehabilitation process is of paramount importance. This enables clinicians to tailor interventions with greater precision to the patient's stage of recovery and to formulate more personalized return‐to‐sport recommendations. Consequently, this study aimed to investigate the cortical and neuromuscular activity of individuals within the first year following ACL reconstruction. In more detail, the neuromuscular and cortical activity during a JPS test, as well as the neuromuscular activity during stair descent and single‐leg hop for distance landings, were the focus of measurement at specific time points between 1.5 and 12 months following ACL reconstruction. It was hypothesized that neuromuscular activity of the quadriceps muscle would be reduced in individuals following ACL reconstruction compared to healthy controls, while hamstring muscle activity would be increased [22, 35, 36]. Furthermore, it was hypothesized that there would be an increase in frontal theta power and a decrease in parietal alpha‐2 power among individuals who had undergone ACL reconstruction, in comparison to healthy controls [27, 28].

## 2 Material and Methods

### 2.1 Participants

In this prospective observational study, 31 participants who had undergone ACL reconstruction were recruited in collaboration with their respective orthopedic surgeons between March 2022 and September 2023. Additionally, 31 healthy controls volunteered to participate in the study. All measurements were conducted at the Bern Movement Lab, Bern University of Applied Sciences, Switzerland. The a priori sample size calculation based on the effect size of 0.799 as determined by a pilot study [35], an alpha level of 0.05 and 95% power, resulted in a theoretical sample of *n* = 16 per group. The participants were matched according to the following criteria: sex, age, height, mass, and leg dominance. The dominant leg was determined based on the participant's self‐reported preferred leg for kicking a ball [37]. The general inclusion criteria were: age between 18 and 50 years, being physically active (at least two sessions of 45 min per week) either currently for the healthy control group or before the ACL injury for the ACL group. Furthermore, it was an exclusion criterion for participants in both groups that they had previously suffered from knee pathologies. The ACL group was required to have a clinically confirmed ACL rupture and to have undergone reconstructive surgery utilizing a quadriceps tendon graft within 8 weeks of the injury. Concomitant injuries were not a limiting factor. Individuals with cardiac, neurological, or peripheral vascular diseases; acute infections; alcohol abuse; current pain medication; other lower extremity or trunk injuries; back pain; thrombosis; pregnancy; dementia; or any other musculoskeletal disorders that would limit their ability to complete the test protocol were excluded from participation. The study was preregistered (DRKS‐ID: 00023002), conducted in accordance with the principles of the Declaration of Helsinki and received ethical approval from the local authority, the “Kantonale Ethikkommission für die Forschung” (KEK Bern, CH, No. 2020–02200). Prior to their participation in the study, all participants provided written informed consent.

### 2.2 Procedure

The ACL group was invited to participate in a series of measurements at five specific time points: approximately 1.5 months (M1), 3–4 months (M2), 6 months (M3), 9 months (M4) and 12 months (M5) postreconstructive surgery. The healthy control group was invited to participate in a single assessment. Once the inclusion criteria had been confirmed, data on anthropometrics, leg dominance, the side of ACL rupture and reconstruction, and any associated injuries in the ACL group were recorded. Additionally, the Tegner Activity Score (TAS) [38] (pre‐injury for the ACL group) and the current Knee Osteoarthritis Outcome Score (KOOS) [39] were collected at the initial and final measurement time point to characterize the participants' pre‐injury activity level and current knee‐related function. These measures were included for descriptive purposes only and were not considered as primary or secondary outcome variables in the analysis. The ACL group also provided information on whether they received preoperative rehabilitation or details of their current rehabilitation program at each measurement time point.

Subsequently, the participants were prepared for the recording of the cortical activity using electroencephalography (EEG; see EEG Recordings and Processing) and the neuromuscular activity using electromyography (EMG; see EMG Recordings and Processing). After the preparation, participants completed a 5‐min treadmill walk at a pace of 3 km/h (Quasar med, h/p/cosmos sports & medical GmbH, Nussdorf‐Traunstein, Germany) as a standardized warm‐up, with neuromuscular activity recorded during the final minute for later submaximal normalization of the EMG signals [35]. Following this, resting EEG activity was recorded during a 3‐min seated rest with eyes open and was later used for EEG signal normalization [40].

**FIGURE 1:**
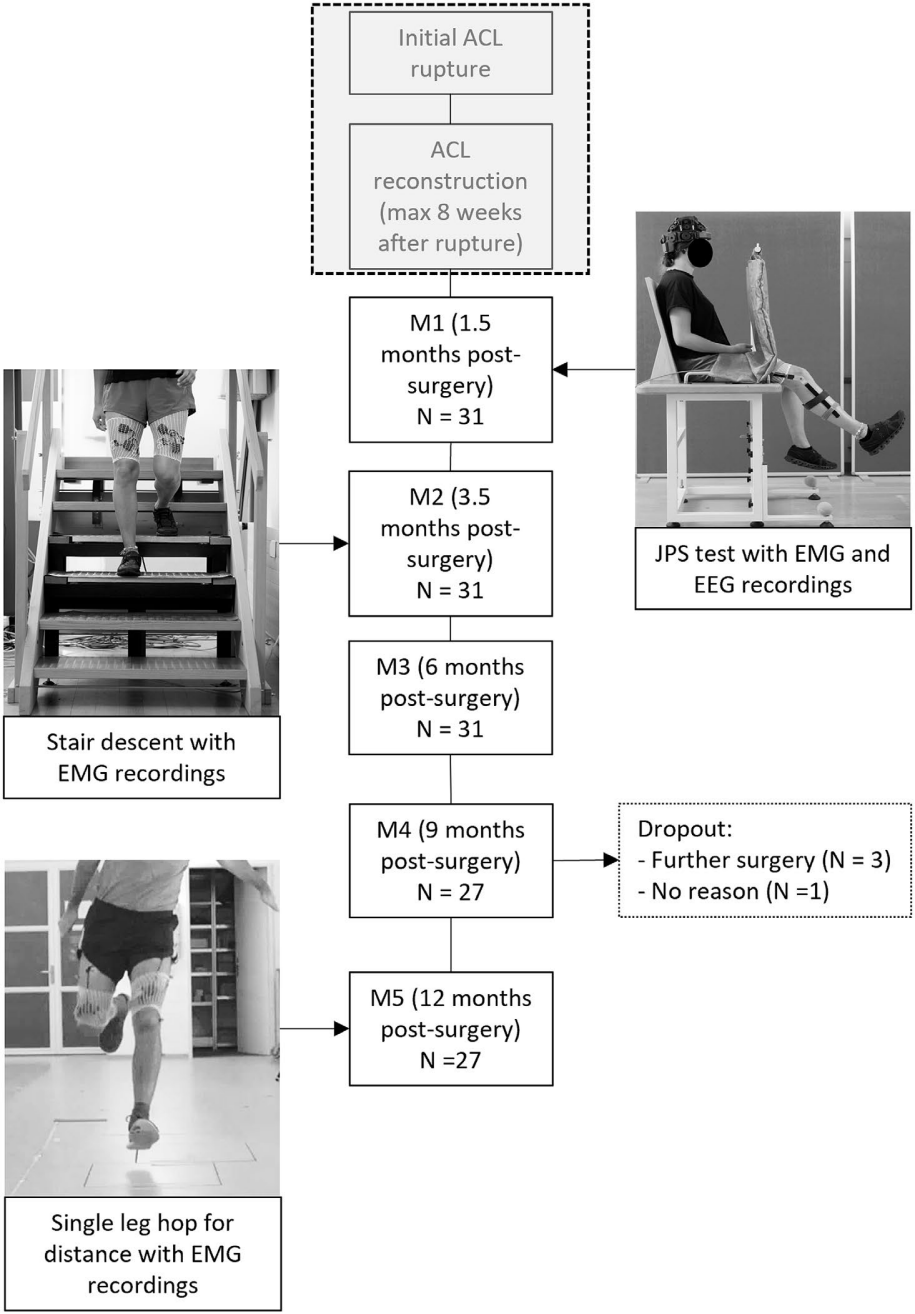
Study flow‐chart representing the timeline, number of participants and included tests. After the implementation, the tests were carried out at each of the following measurement time points. EEG, electroencephalography; EMG, electromyography; JPS, joint position sense; M, measurement time point; *N*, number.

Afterwards, the participants proceeded with the movement tasks. At each measurement time point (M1‐M5), the participants performed an active JPS test in the knee, with simultaneous recordings of the angular changes, along with cortical electrical and neuromuscular activity. From the second measurement time point (M2) onwards, the participants completed stair descent with recordings of neuromuscular activity and at the last measurement time point (M5), a series of single‐leg hop for distance with recording of the neuromuscular activity and jump distance was added (Figure 1).

The JPS test is described in detail elsewhere [41, 42]. The procedure is summarized as follows: during five familiarization trials, the participants actively flexed their knee to 90° (0° = full extension) and extended it to a target angle of 50°, guided by the instructions provided by the examiner and visual feedback on a screen, although the participants' view of their legs was obstructed by a hanging curtain [41]. Testing phase comprised two 3‐min blocks per leg without visual feedback, at a self‐selected pace and the leg order was randomized. Throughout the JPS test, the reproduced angle, along with neuromuscular and cortical electrical activity were recorded. For the recordings of the reproduced angle, an electrogoniometer (Potentiometer RP20, Megatron Elektronik GmbH & Co.KG, Munich, Germany) [41]. The signals of the electrogoniometer were collected using Imago Record software with a sampling rate of 4000 Hz and further processed using Imago Process Master (Pfitec, Endingen, Germany). The angular error, defined as the discrepancy between the targeted and reproduced angles, was calculated as constant angular error (CE) and absolute angular error (AE) [43].

The stair descent task required participants to descend a custom‐designed wooden staircase comprising six steps [35, 44], completing ten repetitions at a self‐determined pace and without the use of handrails. Two force plates (type 9286BA, Kistler Instrumente AG, Winterthur, CH) were embedded in the third and fourth steps to record ground reaction forces for later movement phase definition.

The final task was the single‐leg hop for distance [45, 46, 47]. The participants were instructed to commence each jump on one leg and jump as far as possible, accelerating themselves with the movements of the arms and lower limb joints. They were required to land on the same leg on an embedded force plate (OR6, AMTI Force and Motion, Watertown, MA, USA) and maintain their balance for a period of 12 s. If the participants failed to accomplish the requirements, the trial was repeated. The participants were permitted to select their starting distance, with the option to undertake as many practice trials as necessary on each leg until they felt confident in their ability to perform the jumps. The starting leg was randomly selected. The neuromuscular activity and jumping distance of three valid trials per limb were recorded. The limb symmetry index (LSI) for the ACL group was calculated based on the jump distances in each limb.

### 2.3 EEG Recordings and Processing

The electrical cortical activity was recorded continuously throughout each JPS testing block using a dry EEG system (DSI‐24, Wearable Sensing, San Diego, CA, USA) with a software (DSI‐STREAMER V.1.08.44) operating at 300 Hz. The dry‐EEG headset comprised 19 electrodes positioned according to the 10:20 system and two electrodes on the earlobes [48]. The impedances were maintained below 5 kΩ. The signals were processed in MATLAB (R2020a, Mathworks Inc., Natick, MA, USA) using EEGLAB (eeglab2022.1) [49], following a standardized pipeline [42]. The two blocks of JPS testing per leg were combined, line noise was removed via the CleanLine plugin [50], and the signals were band‐pass filtered (1–30 Hz). Artifact Subspace Reconstruction [51] was employed to correct erroneous data, followed by manual rejection of artifacts. Adaptive mixture independent component analysis [52] was applied to eliminate nonbrain signals like muscle activity, eye movements and electrocardiogram interference. The data were rereferenced to a common average with channel Pz restored. The power spectra for the theta (4–7.5 Hz) and alpha‐2 (10.5–12.5 Hz) bands were calculated via Fast Fourier Transformation and extracted based on the region of interest. These were defined as (F3, Fz, F4) for the theta frequency band, and parietal (P3, Pz, P4) for the alpha‐2 frequency band based on previous investigations [40]. The spectral power values were normalized to resting EEG for further statistical analysis [42].

### 2.4 EMG Recordings and Processing

For surface EMG measurements, electrodes were placed on the M. vastus medialis (VM), M. vastus lateralis (VL), *M. rectus* femoris (RF), M. biceps femoris (BF) and M. semitendinosus (ST) of both legs, following SENIAM guidelines [53]. Prior to placement, the skin was prepared by shaving, abrasion, and alcohol cleaning to enhance signal detection. Bipolar electrodes (Type P‐00‐S, Blue Sensor, Ambu, Ballerup, Denmark) with a 20 mm interelectrode distance were utilized, and the impedance maintained below 2 kΩ. The wireless transmission of measured values was enabled by utilizing the Myon 320 System (Myon AG, Schwarzenberg, Switzerland), which operates with a sampling frequency of 4000 Hz. The data was then recorded using the Imago Record software [20, 21, 35]. During treadmill‐walking, EMG signals were recorded from all muscles previously described, while during the JPS test, only the above‐mentioned muscle activities of the quadriceps and during stair descent the VM, VL, BF and ST muscle activities were recorded. The rectus femoris was recorded and analyzed only during the JPS test, as this task allowed for a more isolated evaluation of quadriceps activation and to be coherent with the existing literature [27]. These EMG signals were processed using Imago Process Master, with full‐wave rectification and band‐pass filtering (10–500 Hz) applied. During the jump landings the neuromuscular activity of the VM, VL, BF and ST muscles was recorded. For half of the control group, these recordings were conducted using the aforementioned Imago Record software, while the remaining data was collected using Vicon Nexus (Vicon Motion Systems Ltd., Oxford, UK) at a sampling frequency of 1000 Hz, due to technical requirements. The raw signals obtained from the jump landings, regardless of the recording system, were full‐wave rectified and band pass filtered (10–400 Hz). The root mean squares (RMS) of the EMG amplitudes were calculated for each muscle and phase per movement task. The submaximal normalization of neuromuscular activity during JPS testing and stair descent was conducted using the mean gait cycle activity during level walking [35]. As a consequence of the utilization of two recording softwares during jump landings, the data was normalized to the maximum RMS value observed during the landing period.

The neuromuscular activity during the JPS test was analyzed during the extension phase (EXT), from the commencement of the movement until the target angle was reached, based on the data obtained from the electrogoniometer. The neuromuscular activity during the stair descent was divided into three phases: the pre‐activation phase (PRE), which included the 150 ms preceding foot‐floor contact until the point of initial contact; the weight acceptance phase (WA), which commenced at the point of initial contact and continued until the minimum vertical ground reaction force was recorded; and the push‐off phase (PO), which continued until the vertical ground reaction forces reached zero [20]. For the single‐leg hop for distance task, the neuromuscular activity was divided into two phases: the pre‐activation, which was defined as 150 ms prior to initial contact, and the landing phase, comprising the phase from initial contact until 250 ms afterwards based on the initial contact with the floor during landing, which was captured by the force plate. A 150 ms time window was selected due to the influence of muscular pre‐activation on neuromuscular control during jump landings [24, 54]. In line with previous studies, a 250 ms window following initial contact was defined [55, 56]. This is based on the assumption that this time period corresponds to the occurrence of maximal knee flexion during the SLHD, thereby encompassing the phase during which the highest knee joint forces are absorbed [57].

### 2.5 Statistical Analysis

The statistical analysis was conducted using the R software (version 4.3.2, 2023) [58]. Group differences in participant characteristics were tested using a Mann–Whitney U test. For the healthy control group, analyses were conducted on a single‐leg, corresponding to the limb affected by the ACL injury in the matched pair, thereby representing the involved leg. The analysis did not incorporate the factor of sex or limb dominance due to an absence of evidence to suggest that it exerted a significant influence [41, 59, 60]. The results are presented in terms of means, estimated marginal means, standard deviations (SD), and standard errors (SE). The inferential analysis of the JPS test and stair descent concentrated on the independent variable, designated as “Group”, with factors including group (ACL or healthy), measurement time (M1–M5), and limb (involved or noninvolved limb) and the corresponding dependent variables of the movement tasks. A linear mixed model was applied to each dependent variable with the random intercept specified as “Group: subject” [42]. Contrasts of interest were calculated relative to the reference category (matched‐involved leg of healthy controls), with standard errors, 95% CIs, and *p*‐values adjusted using Tukey's method. The lme4 package [61] was used for model fitting, and emmeans package [62] for computing contrasts. The neuromuscular activity during the jump landings phases and distances were evaluated using an independent *t*‐test for the involved limb of the ACL group in contrast to the control group, and a dependent *t*‐test was employed to compute intralimb differences of the ACL group. Effect sizes were calculated as rank‐biserial correlation coefficient (*r*) for each comparison [63] and defined as small (0.1), medium (0.3) and strong (0.5) [64]. To account for the issue of multiple testing, the *p*‐values were adjusted using the Bonferroni method. The datasets and statistical methods employed in this study are publicly accessible via the Olos repository at the following link: https://doi.org/10.34914/olos:ahh46lkzqze6xlb3fnfr6cutuu [65].

## 3 Results

### 3.1 Participants

In total 62 participants took part in this study including 31 participants following ACL rupture and reconstruction. The flow chart of the study protocol and the numbers of participants per measurement time point can be found in Figure 1. Participant's characteristics are presented in Table 1. Details on the associated injuries, rehabilitation content and frequency per measurement time point of the ACL group can be found in the Supporting Information (see Tables S1 and S2).

**TABLE 1:** Characteristics of the participants displayed as mean ± standard deviation and between group comparison using Mann–Whitney *U* tests.

Characteristic	ACL (*n* = 31)	Control (*n* = 31)	*p*
Age (years)	25.6 ± 6.2	26.9 ± 6.2	0.33
Height (cm)	173.5 ± 9.1	175.5 ± 9.3	0.37
Mass (kg)	72.0 ± 11.8	70.7 ± 10.6	0.72
Female sex (*n*; %)	12; 38.7%	12; 38.7%	—
Tegner score (10 points max)	Pre‐injury = 6 ± 1.5	4.9 ± 1	ACL Pre‐injury vs. Con = 0.007
	M5 = 5.6 ± 1.4		ACL‐M5 vs. Con = 0.12
Total KOOS score (168 points max)	M1 = 89 ± 23.6	166 ± 3.2	ACL‐M1 vs. Con < 0.001***
	M5 = 146.8 ± 12.7		ACL‐M5 vs. Con < 0.001***
Pain (36 points max)	M1 = 20.6 ± 6.5	35.7 ± 0.8	ACL‐M1 vs. Con < 0.001***
	M5 = 32 ± 3.5		ACL‐M5 vs. Con = 0.005**
Symptoms (28 points max)	M1 = 13.1 ± 4.2	26.9 ± 1	ACL‐M1 vs. Con < 0.001***
	M5 = 21.7 ± 3.6		ACL‐M5 vs. Con < 0.001***
ADL (68 points max)	M1 = 47.2 ± 12.3	67.9 ± 0.3	ACL‐M1 vs. Con < 0.001***
	M5 = 65.7 ± 3.5		ACL‐M5 vs. Con = 0.5
Sport and Recreation (20 points max)	M1 = 3.4 ± 3.3	19.7 ± 0.7	ACL‐M1 vs. Con < 0.001***
	M5 = 16.6 ± 2.5		ACL‐M5 vs. Con < 0.001***
KRQoL (16 points max)	M1 = 4.7 ± 2.2	15.7 ± 1.3	ACL‐M1 vs. Con < 0.001***
	M5 = 10.5 ± 2.4		ACL‐M5 vs. Con < 0.001***
Single leg hop distance (cm)	M5‐inv = 111.9 ± 28.2	123.6 ± 35.8	ACL‐M5‐inv vs. CON = 0.20
	M5‐noninv = 123.5 ± 28.8		ACL‐M5‐inv vs. ACL‐M5‐noninv = < 0.001***

*Note:* Asterisks indicate statistical significance (**p < 0.01, ***p < 0.001).

Abbreviations: ACL, anterior cruciate ligament group; ADL, Activities of daily living; inv, involved limb; KOOS, Knee injury and Osteoarthritis Outcome Score; KRQoL, knee‐related quality of life; M1/5, measurement time point 1/5; noninv, noninvolved limb.

### 3.2 Joint Position Sense Test

The descriptive statistics of the constant and absolute error are displayed in Table 2. The matched involved limb of the control group revealed a mean constant error of 11.0° with a standard deviation of 7.7° and an absolute error of 11.5° ± 7.0°.

**TABLE 2:** Descriptive statistic of the constant and absolute error of the JPS test of the ACL group per measurement time point and limb.

Variable	Time point and limb	ACL (Mean + SD)
Constant error [°]	M1‐inv	6.2 ± 5.4
	M1‐noninv	7.4 ± 5.8
	M2‐inv	6.1 ± 4.9
	M2‐noninv	5.4 ± 6.0
	M3‐inv	6.1 ± 5.8
	M3‐noninv	5.3 ± 5.4
	M4‐inv	4.0 ± 5.0
	M4‐noninv	4.2 ± 4.9
	M5‐inv	4.3 ± 4.5
	M5‐noninv	4.7 ± 5.0
Absolut error [°]	M1‐inv	6.9 ± 4.4
	M1‐noninv	8.0 ± 4.9
	M2‐inv	6.6 ± 4.2
	M2‐noninv	6.6 ± 4.7
	M3‐inv	6.9 ± 4.8
	M3‐noninv	6.2 ± 4.3
	M4‐inv	5.1 ± 3.8
	M4‐noninv	5.3 ± 3.7
	M5‐inv	5.0 ± 3.6
	M5‐noninv	5.6 ± 4.0

Abbreviations: ACL, anterior cruciate ligament group; inv, involved limb; M1‐5, measurement time point 1–5; noninv, noninvolved limb; SD, standard deviation.

The absolute error was found to be statistically significantly smaller in both limbs of the ACL group compared to the healthy control group at all measurement time points (*p* < 0.004, Table 3).

**TABLE 3:** Linear mixed model output for the absolute error of the JPS test with Group as fixed effect and Group:Subject as random effect with random intercept. Control group (involved limb) served as the reference category (absolute error = 11.5 ± 7.0).

ACL time point and limb	Absolute error model output		
	Beta	SE	*p*
M1‐inv	−4.18	0.88	< 0.001***
M1‐noninv	−3.12	0.87	0.004**
M2‐inv	−4.60	0.87	< 0.001***
M2‐noninv	−4.65	0.87	< 0.001***
M3‐inv	−4.24	0.87	< 0.001***
M3‐noninv	−4.88	0.87	< 0.001***
M4‐inv	−5.53	0.9	< 0.001***
M4‐noninv	−5.78	0.9	< 0.001***
M5‐inv	−5.94	0.9	< 0.001***
M5‐noninv	−5.56	0.91	< 0.001***

*Note:* Asterisks indicate statistical significance (**p < 0.01, ***p < 0.001).

Abbreviations: ACL, anterior cruciate ligament group; inv, involved limb; M1‐5, measurement time point 1–5; noninv, noninvolved limb; SE = standard error.

Neuromuscular activity during extension phase was significantly reduced in the VL of the noninvolved limb of the ACL group at 1.5 months (−42%, *p* < 0.001), 3–4 months (−28.5%; *p* = 0.005), 6 months (−27.0%; *p* = 0.011) and 9 months (−21.9%; *p* = 0.048) following surgery, when compared to the healthy control group. The VL of the involved limb exhibited a statistically significant reduction (−27.2%; *p* = 0.012) 9 months postsurgery when compared to the healthy control group. No significant differences were identified in the VM or RF (Table 4).

**TABLE 4:** Descriptive statistic of the normalized neuromuscular activity during extension to the target angle and linear mixed model output with Group as fixed effect and Group:Subject as random effect with random intercept. Control group (involved limb) served as the reference category (Control group involved limb: Vastus medialis RMS [% subMVC] = 84.1 ± 55.3; vastus lateralis RMS [% subMVC] = 94.1 ± 62.6; rectus femoris RMS [% subMVC] = 111.0 ± 64.6).

Variable	ACL‐group		Model output		
	Time point and limb	Mean ± SD	Beta	SE	*p*
Vastus medialis RMS [% subMVC]	M1‐inv	100.5 ± 54.1	12.1	11.1	0.822
	M1‐noninv	66.3 ± 32.3	−22	11.1	0.28
	M2‐inv	79.7 ± 43.9	−5.5	11	0.988
	M2‐noninv	73.3 ± 32.1	−18.6	11.1	0.463
	M3‐inv	78.8 ± 34.7	−10.5	11	0.885
	M3‐noninv	75.0 ± 32.2	−13.3	11	0.767
	M4‐inv	78.7 ± 37.8	−11.9	11.4	0.846
	M4‐noninv	72.7 ± 35.3	−17.6	11.5	0.558
	M5‐inv	83.1 ± 51.6	−10.5	11.3	0.895
	M5‐noninv	80.0 ± 38.5	−9.2	11.6	0.937
Vastus lateralis RMS [% subMVC]	M1‐inv	94.5 ± 53.0	−10.5	12	0.916
	M1‐noninv	55.5 ± 22.6	−52.7	11.9	< 0.001***
	M2‐inv	92.2 ± 55.2	−18.2	11.8	0.561
	M2‐noninv	67.2 ± 35.4	−41	11.8	0.005**
	M3‐inv	81.8 ± 42.7	−29.7	11.8	0.095
	M3‐noninv	68.5 ± 34.1	−38.2	11.8	0.012*
	M4‐inv	68.6 ± 27.3	−39.4	12.2	0.013*
	M4‐noninv	73.5 ± 33.3	−33.8	12.2	0.050
	M5‐inv	77.4 ± 45.2	−33.1	12.3	0.058
	M5‐noninv	77.8 ± 46.8	−25.8	12.4	0.241
Rectus femoris RMS [% subMVC]	M1‐inv	99.4 ± 47.9	−14.2	11.2	0.739
	M1‐noninv	103.4 ± 58.1	−17.2	11.2	0.570
	M2‐inv	108.3 ± 51.9	−13.9	11.2	0.755
	M2‐noninv	104.4 ± 45.1	−14.9	11.1	0.694
	M3‐inv	102.3 ± 45.3	−16.6	11.2	0.607
	M3‐noninv	100.1 ± 41.9	−18.9	11.1	0.462
	M4‐inv	103.8 ± 43.0	−15.9	11.5	0.672
	M4‐noninv	98.6 ± 40.2	−19	11.7	0.507
	M5‐inv	101.6 ± 39.5	−13.7	11.6	0.783
	M5‐noninv	101.4 ± 53.0	−16.3	11.8	0.658

*Note:* Asterisks indicate statistical significance (*p < 0.05, **p < 0.01, ***p < 0.001).

Abbreviations: ACL, anterior cruciate ligament; inv, involved limb; M1‐5, measurement time point 1–5; noninv, noninvolved limb; RMS, root mean square; SD, standard error; SE, standard error; subMVC, submaximal voluntary contraction.

No significant differences were observed in the electrical cortical activity within the theta frequency band over the frontal electrodes and the alpha‐2 frequency band over the parietal electrodes between the ACL and the healthy control group. Given the absence of significant findings and to maintain clarity of the main manuscript, detailed EEG results are reported in the supporting material (see supplemental Table S3).

### 3.3 Stair Descent

**FIGURE 2:**
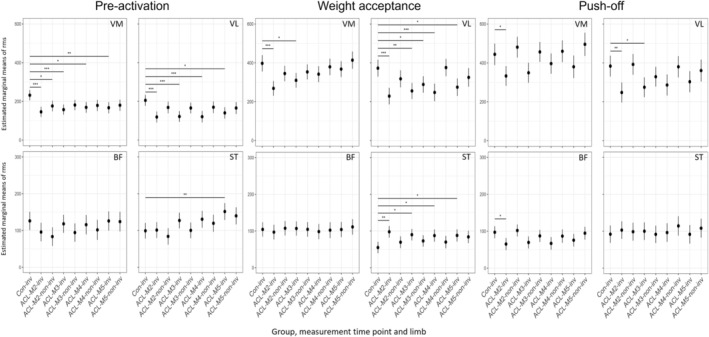
Neuromuscular activity during the pre‐activation, weight acceptance and push‐off phase of the stair descent. Estimated marginal means (black point) with 95% confidence intervals are resulting from linear mixed model analysis with Group as fixed effect and Group: Subject as random effect with random intercept are presented. Significant differences are displayed with * for *p* < 0.05, ** for *p* < 0.01 and *** for *p* < 0.001. Cave: To enhance readability, the *y*‐axis was adjusted in the lower row of the graph. It is not recommended to make direct visual comparisons. BF, biceps femoris; inv., involved; M2‐5, measurement time point 2–5; noninv, noninvolved; rms, root mean square; ST, semitendinosus; VL, vastus lateralis; VM, vastus medialis.

During PRE of the stair descent, a significant reduction of 26% to 43% in the neuromuscular activity of the VM and VL of the involved limb was observed at all measurement time points (0.001 < *p* < 0.01 and 0.0002 < *p* < 0.007, respectively), when compared to the control group. Furthermore, the neuromuscular activity of the VM of the noninvolved limb was found to be significantly reduced by 19% in comparison to the control group at 3–4 months postsurgery (*p* = 0.029). The ST exhibited a significantly elevated neuromuscular activity of 49% in the involved limb of the ACL group in comparison to the control group at 12‐month follow‐up (*p* = 0.004), while the BF did not differ significantly (Figure 2; see supplemental Table S4).

During WA, the neuromuscular activity of the VM in the involved limb was found to be significantly reduced by 32% at 3–4 months (*p* < 0.001) and by 24% at 6 months (*p* = 0.018) following surgery compared to the control group. A comparison with the control group revealed a significant reduction with a range of 20% to 35% in the neuromuscular activity of the VL of the involved limb at all measurement time points (0.001 < *p* < 0.016). Additionally, a significant reduction of 18% was observed in the VL of the noninvolved leg of the ACL group at 6 months postsurgery (*p* = 0.045). In comparison to the control group, the neuromuscular activity of the ST of the involved limb of the ACL group exhibited a significant increase with a range of 62% to 80% at all measurement time points (0.001 < *p* < 0.018). No significant differences were observed in the BF comparison (Figure 2; see supplemental Table S4).

During PO, the neuromuscular activity of the VM in the involved limb of the ACL group was significantly reduced by 25% at 3–4 months following surgery (*p* = 0.025) in comparison to the control group. The VL demonstrated a significantly diminished level of neuromuscular activity, of 40% in the involved limb compared to the control group at both 3–4 months (*p* = 0.003) and 34% at 6 months (*p* = 0.028) postsurgery measurement time points. A significant reduction of 31% in the neuromuscular activity of the BF was observed in the involved leg of the ACL group when compared to the control group at 3–4 months following surgery (*p* = 0.047). No significant differences were identified in the ST during the PO phase (Figure 2; see supplemental Table S4).

### 3.4 Single‐Leg Hop for Distance

Two participants were unable to complete the single‐leg hop test due to current pain or insufficient confidence to perform the test, leaving 25 ACL participants included in this analysis. Single‐leg hop distances of the involved limb of the ACL group were found to be significantly less in comparison to the contralateral leg (*p* < 0.001). No significant difference was observed between the involved and matched control limb (*p* = 0.20) (Table 1). The limb symmetry index (LSI) ranged from 59% to 111% in the ACL group with eleven participants below 90% (LSI); details are shown in the supplementary file (see supplemental Table S5).

During the pre‐activation phase of the jump landings, the neuromuscular activity of the ST was found to be significantly reduced by 19% (*p* < 0.001) in the involved limb in comparison to the control group, with medium effect size. A significant reduction in the neuromuscular activity of the VM and VL, with medium to strong effect sizes, was observed in the noninvolved limb compared to the involved limb (−27% and −19%; *p* < 0.001 and *p* = 0.002, respectively). No other comparisons yielded statistically significant results during the pre‐activation phase (Table 5). During the landing phase, a significant reduction in neuromuscular activity was observed in all recorded muscles when comparing the ACL involved limb with the control group (−20% to −29%; *p* ≤ 0.001), accompanied with medium to strong effect sizes. A significant elevation in neuromuscular activity of the VL in the noninvolved limb, with medium effect size was found in the within‐group comparison of the ACL participants (+6%; *p* = 0.017; Table 5).

**TABLE 5:** Descriptive statistics and between and within group comparisons (independent and dependent *t*‐tests, respectively) of the normalized neuromuscular activity during single leg hop for distance landings.

		ACL‐involved limb (1)	ACL‐noninvolved limb (2)	Control involved limb (3)	(1) vs. (2)	(1) vs. (3)
		Mean ± SD	Mean ± SD	Mean ± SD	*p*‐value effect size	*p*‐value effect size
PRE‐ACTIVATION RMS [% max RMS]	Vastus medialis	37.4 ± 12.8	27.5 ± 9.6	38.8 ± 15.7	*p* < 0.001*** *r* = 0.6	*p* = 0.916 *r* < 0.1
	Vastus lateralis	43.7 ± 15.7	35.7 ± 16.8	50.1 ± 22.4	*p* = 0.002** *r* = 0.4	*p* = 0.08 *r* = 0.1
	Biceps femoris	67.4 ± 18.6	65.1 ± 24.2	74.9 ± 38.5	*p* = 0.634 *r* = 0.1	*p* = 0.345 *r* = 0.1
	Semitendinosus	75.6 ± 21.7	68.7 ± 26.6	93.6 ± 29.5	*p* = 0.222 *r* = 0.2	*p* < 0.001*** *r* = 0.3
LANDING RMS [% max RMS]	Vastus medialis	59.1 ± 11.6	61.7 ± 11.7	73.6 ± 15.9	*p* = 0.144 *r* = 0.2	*p* < 0.001*** *r* = 0.4
	Vastus lateralis	60.2 ± 11.8	64.2 ± 13.6	76.4 ± 16.6	*p* = 0.017* *r* = 0.3	*p* < 0.001*** *r* = 0.5
	Biceps femoris	46.6 ± 14.6	49.6 ± 16.6	60.9 ± 25.2	*p* = 0.300 *r* < 0.1	*p* = 0.001*** *r* = 0.3
	Semitendinosus	46.7 ± 17.3	45.5 ± 15.2	65.5 ± 25.0	*p* = 0.812 *r* = 0.2	*p* < 0.001*** *r* = 0.4

*Note:* Asterisks indicate statistical significance (*p < 0.05, **p < 0.01, ***p < 0.001).

Abbreviations: ACL, anterior cruciate ligament group; RMS, root mean square; SD, standard deviation.

## 4 Discussion

The study presents notable findings regarding neuromuscular and cortical activity during the initial year following ACL reconstruction, offering valuable insights for further research and potential modifications to clinical practice. The performance outcomes of the JPS test were superior in the ACL group, whereas the single‐leg hop distance did not differ between groups but within the ACL group. The results demonstrated an alteration in neuromuscular activity in the ACL group at all measurement time points and tasks. The electrical cortical activity was unaltered in comparison to the healthy control group.

### 4.1 JPS Test

JPS errors were significantly lower in the ACL group at all measurement points. This finding is at odds with the existing literature reporting higher or equal JPS errors in the ACL group [66, 67, 68]. This phenomenon may be attributable to the ACL group's exclusive focus on knee rehabilitation, stemming from the severe injury, and their regular engagement in movement correction and rehabilitation, especially in the early measurement time points. This is not the case in the healthy control group, who may not pay attention to their knee during the execution of the task. Moreover, the duration of the JPS test may have led to further indifference or boredom among the control group. The reduction in neuromuscular activity, mainly observed in the VL of the noninvolved limb during the extension to the target angle, is consistent with the findings of bilateral consequences after unilateral ACL reconstruction [20, 69]. The observed alterations in neuromuscular activity may have been the consequence of altered central processing. However, the present study did not identify any statistically significant differences between the ACL and control groups with regard to the electrical cortical activity observed during the JPS test. This differs from the findings of Baumeister et al., who reported a significant increase in theta power over frontal and a decrease in alpha‐2 power over parietal cortex areas [27]. Nevertheless, an MRI study indicated that there were no significant differences in brain activity during a JPS test in participants following ACL reconstruction, which is supported by the present findings [70]. The absence of significant EEG differences should be interpreted with caution. It could be argued that the open‐chain JPS test provided an insufficiently challenging environment for physically active young participants. It may be beneficial to perform the test as a more functional task in order to elicit higher demands on the sensorimotor system. Additionally, the inherent variability of EEG measures and the relatively small sample size may have reduced sensitivity to detect subtle effects.

### 4.2 Stair Descent

During descent of the stairs, altered neuromuscular activation was observed above all in the involved limb at all measurement time points, which is in accordance with the findings of previous studies [14, 20]. The interplay of decreased quadriceps and increased hamstrings neuromuscular activity may be a consequence of the process of athrogenic muscle inhibition (AMI) [71]. In the early stages, this can be a preventive strategy to elicit greater knee stability and prevent higher ACL strains. Nevertheless, the present study demonstrates that this neuromuscular function is not restored during the standard rehabilitation process, which may contribute to the differences in joint forces which could lead to the early onset of knee osteoarthritis [72]. The existence of these differences in everyday tasks, as demonstrated by the outcomes observed during stair descent, necessitates a reconsideration of rehabilitation content to prevent joint degeneration. In recent years, recommendations have been made regarding the incorporation of neuromuscular training and relearning of motor skills following ACL rupture [73, 74, 75]. The findings of the present study emphasize the importance of further research and focus on the implementations to facilitate a comprehensive rehabilitation for patients with ACL ruptures and reconstructions.

### 4.3 Single‐Leg Hop for Distance

Neuromuscular activity during the landing phase following single‐leg hops was found to be significantly reduced in all assessed muscles in the ACL involved limb. This is in contrast to the elevated neuromuscular activity in the hamstring muscles, which was observed during the stair descent. It can be hypothesized that the reduction of neuromuscular activation in the hamstring muscles during the landing contributes significantly to the elevated injury risk by failing to provide protective muscle stiffness to reduce potential shear forces acting on the ACL [76]. A more in‐depth analysis of thigh‐hamstring muscle coactivation may provide further insights relevant to reaching a comprehensive conclusion. Moreover, the pre‐activations were significantly altered in the quadriceps in the contralateral limb. It can be hypothesized that this could lead to an increased risk of injury to the contralateral limb, as it has been documented in the extant literature [11].

Although the landing was performed in a controlled and laboratory setting, the instructions given were similar to those given in a sporting context, namely to jump as far as possible. This could have resulted in a shift of focus on the task, which may have contributed to the deviations in neuromuscular activity. Besides no statistical differences in jumping distance were observed between the involved limb of the ACL group and the healthy control group, a considerable number of participants following ACL reconstruction were unable to reach a 90% LSI, which reflects an ongoing functional deficit. Furthermore, this observation highlights the heterogeneity in recovery following ACL reconstruction, thereby emphasizing the importance of individualized rehabilitation. In addition to the aforementioned recommendations for rehabilitation, it is important to note that rehabilitation content may need to be adapted to a sporting context in order to enhance an individual's return to competition readiness [77]. This contributes to the discussion of further reconsideration of the RTS test battery [78]. It has been proposed that the existing RTS test battery should be expanded to include, if possible, neuromuscular measurements during high‐risk situations (e.g., side‐cutting maneuvers) [79] or neurocognitive testing (e.g., dual‐task conditions) [77] in order to assess functional performance under more realistic, everyday life settings. It could be hypothesized that these assessments could lead to a decline in the occurrence of subsequent injuries, attributable to an enhanced RTS decision‐making process [77].

## 5 Limitations

There are some limitations to be considered. The participants who had undergone ACL reconstruction were permitted to have concomitant injuries but were restricted to a single surgical procedure. This limits the generalizability of the findings to other injury compositions and surgical techniques. Moreover, any direct translation to conservatively treated ACL ruptures should be conducted with caution and demands for further research. Nonetheless, the present study provides valuable insights from a typical rehabilitation process, which is likely to remain consistent regardless of the ACL surgical technique employed. It should be noted that the rehabilitation content and frequency were not controlled among the ACL participants, which could have impacted the results and further limits the generalizability to other health care systems.

The self‐selected pace adopted during the JPS test and stair descent may have influenced the recorded EMG signals. The use of a metronome may facilitate greater standardization of these tasks. However, this may impede the feasibility of the task performance, particularly at the early measurement time points.

Furthermore, the participants were permitted to select their own jumping distance. Although the instruction was to jump as far as possible, it is possible that the participants may have chosen smaller distances in order to perform well. Nevertheless, the distances jumped by the contralateral limb of the ACL group and the healthy controls were similar, indicating that this factor had potentially a negligible influence.

## 6 Perspective

In conclusion, this study provides important longitudinal evidence that neuromuscular alterations persist throughout the first postoperative year following ACL reconstruction, while cortical activity during joint position testing remains statistically unchanged. These findings extend prior research by demonstrating their presence across a variety of tasks and over a number of time points, especially in the early stages, thus filling a knowledge gap [34]. The persistent presence of these impairments emphasizes the limitations of contemporary rehabilitation protocols in adequately addressing underlying sensorimotor deficits that are associated with an elevated risk of reinjury. Consequently, it may be advisable to incorporate targeted neuromuscular and motor relearning interventions, in addition to more comprehensive assessments, into rehabilitation and return‐to‐sport decision‐making processes.

## Funding

This study was funded by the Swiss National Science Foundation (Project No: 215684). The funder played no role in the data collection, analysis, interpretation or publication of the finished manuscript.

## Conflicts of Interest

The authors declare no conflicts of interest.

## Supporting information


**Table S1:** Main content and frequency of the rehabilitation following ACL reconstruction on the respective time points. Information on the content and frequency was provided by the participants at the respective measurement time points.
**Table S2:** Number of participants with corresponding associated injuries.
**Table S3:** Descriptive statistic of the normalized electrical cortical activity during joint position sense test and linear mixed model output with Group as fixed effect and Group: subject as random effect with random intercept. Control group (involved limb) served as the reference category (Control group: frontal theta cortical activity [% resting EEG] = 96.2 ± 51.6; parietal alpha‐2 cortical activity [% resting EEG] = 90.7 ± 36.7).
**Table S4:** Descriptive statistic of the normalized neuromuscular activity during the pre‐activation, weight acceptance and push‐off phase of the stair descent and linear mixed model output with Group as fixed effect and Group: subject as random effect with random intercept. Control group (involved limb) served as the reference category (Control group involved limb: Pre‐activation: vastus medialis RMS [% subMVC] = 213.9 ± 126.0; vastus lateralis RMS [% subMVC] = 201.0 ± 116.3; biceps femoris RMS [% subMVC] = 118.7 ± 112.8; semitendinosus RMS [% subMVC] = 99.3 ± 92.1; *Weight acceptance*: vastus medialis RMS [% subMVC] = 386.3 ± 166.3; vastus lateralis RMS [% subMVC] = 347.6 ± 159.5; biceps femoris RMS [% subMVC] = 103.6 ± 88.7; semitendinosus RMS [% subMVC] = 53.9 ± 50.4; *Push‐off*: vastus medialis RMS [% subMVC] = 426.3 ± 168.6; vastus lateralis RMS [% subMVC] = 386.8 ± 179.7; biceps femoris RMS [% subMVC] = 95.5 ± 82.7; semitendinosus RMS [% subMVC] = 90.4 ± 79.1).
**Table S5:** Single‐leg hop distance and limb symmetry index of the ACL group.

## Data Availability

The data that support the findings of this study are openly available in Olos at https://doi.org/10.34914/olos:ahh46lkzqze6xlb3fnfr6cutuu.
