# Supplementary material for: Examining Neuromuscular and Cortical Activity Over 1 Year Following Anterior Cruciate Ligament Reconstruction
Source: Scand J Med Sci Sports. 2026 Aug 11;36(8):e70358. doi: 10.1111/sms.70358 (PMC13459745; doi:10.1111/sms.70358)
Supplement: Supplementary file 1 [file SMS-36-e70358-s001.docx]

**TABLE S1** Main content and frequency of the rehabilitation following ACL reconstruction on the respective time points. Information on the content and frequency was provided by the participants at the respective measurement time points.

| Time point | Main content | Number of participants | Frequency |
| --- | --- | --- | --- |
| Prehabilitation | Mobilisation & massage, low-dose strengthening exercise | 7 | 2-8 appointments over 2-6 weeks |
| 1.5 months post surgery | Mobilisation and low-dose strengthening exercises | 5 | One appointment per week |
|  |  | 23 | Two appointments per week |
|  |  | 3 | Three appointments per week |
| 3.5 months post surgery | Strengthening & balance exercises | 19 | One appointment per week |
|  |  | 10 | Two appointments per week |
|  |  | 2 | Three appointments per week |
| 6 months post surgery | Strengthening & initial plyometrics exercises | 19 | One appointment per week |
|  |  | 4 | Two appointments per week |
|  |  | 8 | One appointment every two weeks |
| 9 months post surgery | Strengthening and plyometrics | 9 | One appointment per week |
|  |  | 10 | One appointment every two weeks |
|  |  | 4 | One appointment per month |
|  |  | 3 | No more appointments |
| 12 months post surgery | Plyometrics and stop-and-go exercises | 5 | One appointment per week |
|  |  | 7 | One appointment every two weeks |
|  |  | 2 | One appointment per month |
|  |  | 12 | No more appointments |

**TABLE S2** Number of participants with corresponding associated injuries.

| Associated injuries | Number of participants |
| --- | --- |
| Lesion of both the medial and lateral meniscus | 7 |
| Lesion of the lateral meniscus | 7 |
| Lesion of the medial meniscus | 6 |
| Lesion of the medial collateral ligament and lateral meniscus | 3 |
| Complete rupture of the medial collateral ligament and lesion of the lateral meniscus | 1 |
| Cartilage lesion of the lateral tibial plateau and lateral meniscus lesion | 1 |
| No associated injuries | 5 |

**TABLE S3** Descriptive statistic of the normalised electrical cortical activity during joint position sense test and linear mixed model output with Group as fixed effect and Group:subject as random effect with random intercept. Control group (involved limb) served as the reference category (Control group: frontal theta cortical activity [% resting EEG] = 96.2±51.6; parietal alpha-2 cortical activity [% resting EEG] = 90.7±36.7)

| **Variable** | **ACL**^†^**-group** | | **Model output** | | |
| --- | --- | --- | --- | --- | --- |
|  | *Time point and limb* | *Mean±SD*^‡^ | *Beta* | *SE*^§^ | *p-value* |
| **Frontal theta cortical activity [% resting EEG**^§§^**]** | M1^¶^-inv^††^ | 103.1±29.0 | 3.9 | 7.4 | 0.985 |
|  | M1-non-inv^‡‡^ | 110.2±27.7 | 11.5 | 7.4 | 0.551 |
|  | M2-inv | 103.3±29.4 | 4.7 | 7.5 | 0.975 |
|  | M2-non-inv | 102.8±32.2 | 4.5 | 7.4 | 0.976 |
|  | M3-inv | 96.9±33.5 | -3.5 | 7.5 | 0.991 |
|  | M3-non-inv | 102.0±35.3 | 3.1 | 7.6 | 0.994 |
|  | M4-inv | 110.8±37.2 | 12.8 | 7.8 | 0.49 |
|  | M4-non-inv | 107.1±34.5 | 8.5 | 7.7 | 0.823 |
|  | M5-inv | 107.8±40.6 | 8.9 | 7.7 | 0.796 |
|  | M5-non-inv | 110.4±42.8 | 11.4 | 7.7 | 0.608 |
| **Parietal alpha-2 cortical activity [% resting EEG]** | M1-inv | 84.6±33.2 | -5.8 | 8.6 | 0.967 |
|  | M1-non-inv | 85.6±34.0 | -4.9 | 8.7 | 0.982 |
|  | M2-inv | 87.0±29.7 | -4.6 | 8.7 | 0.986 |
|  | M2-non-inv | 87.0±28.9 | -3.8 | 8.6 | 0.992 |
|  | M3-inv | 95.7±30.8 | 4.4 | 8.7 | 0.988 |
|  | M3-non-inv | 102.9±35.2 | 12.4 | 8.8 | 0.643 |
|  | M4-inv | 103.3±41.7 | 11.7 | 9.1 | 0.718 |
|  | M4-non-inv | 98.7±44.8 | 8.7 | 8.9 | 0.882 |
|  | M5-inv | 97.2±43.3 | 6.8 | 8.9 | 0.949 |
|  | M5-non-inv | 105.0±45.6 | 14.1 | 9.1 | 0.55 |

†ACL = anterior cruciate ligament; ‡SD = standard error; §SE = standard error; ¶M1-5 = measurement time point 1-5; ††inv = involved limb; ‡‡non-inv = non-involved limb; §§EEG = electroencephalography

**TABLE S4** Descriptive statistic of the normalised neuromuscular activity during the pre-activation, weight acceptance and push-off phase of the stair descent and linear mixed model output with Group as fixed effect and Group:subject as random effect with random intercept. Control group (involved limb) served as the reference category (Control group involved limb: *Pre-activation*: vastus medialis RMS [% subMVC] = 213.9±126.0; vastus lateralis RMS [% subMVC] = 201.0±116.3; biceps femoris RMS [% subMVC] = 118.7±112.8; semitendinosus RMS [% subMVC] = 99.3±92.1; *Weight acceptance*: vastus medialis RMS [% subMVC] = 386.3±166.3; vastus lateralis RMS [% subMVC] = 347.6±159.5; biceps femoris RMS [% subMVC] = 103.6±88.7; semitendinosus RMS [% subMVC] = 53.9±50.4; *Push-off*: vastus medialis RMS [% subMVC] = 426.3±168.6; vastus lateralis RMS [% subMVC] = 386.8±179.7; biceps femoris RMS [% subMVC] = 95.5±82.7; semitendinosus RMS [% subMVC] = 90.4±79.1)

| **Variable** | | **ACL**^†^ **group** | | **Model output** | | |
| --- | --- | --- | --- | --- | --- | --- |
|  |  | *Time point and limb* | *Mean±SD*^‡^ | *Beta* | *SE*^§^ | *p-value* |
| **PRE-ACTIVATION** | **Vastus medialis RMS**^§§^ **[% subMVC^¶¶^]** | M2^¶^-inv^††^ | 139.0±70.2 | -85.9 | 19 | 0.0001*** |
|  |  | M2-non-inv^‡‡^ | 173.2±74.5 | -55.7 | 19.4 | 0.029* |
|  |  | M3-inv | 149.5±74.0 | -74.5 | 18.8 | 0.0008*** |
|  |  | M3-non-inv | 176.1±75.7 | -50.6 | 19 | 0.054 |
|  |  | M4-inv | 156.3±77.8 | -62.8 | 19.6 | 0.010** |
|  |  | M4-non-inv | 171.6±78.3 | -52.8 | 19.9 | 0.055 |
|  |  | M5-inv | 161.9±83.5 | -64.7 | 19.9 | 0.009** |
|  |  | M5-non-inv | 175.1±90.2 | -51.8 | 20.0 | 0.064 |
|  | **Vastus lateralis RMS [% subMVC]** | M2-inv | 118.7±67.5 | -86.1 | 20.3 | 0.003** |
|  |  | M2-non-inv | 160.3±84.1 | -36.7 | 20.9 | 0.375 |
|  |  | M3-inv | 114.9±79.6 | -83.4 | 20 | 0.0004*** |
|  |  | M3-non-inv | 161.3±93.0 | -39.5 | 20 | 0.261 |
|  |  | M4-inv | 118.4±78.7 | -84.6 | 20.7 | 0.0005*** |
|  |  | M4-non-inv | 161.1±93.4 | -35.6 | 20.9 | 0.408 |
|  |  | M5-inv | 138.2±81.0 | -65.0 | 20.8 | 0.014* |
|  |  | M5-non-inv | 158.2±94.9 | -39.7 | 21.2 | 0.308 |
|  | **Biceps femoris RMS [% subMVC]** | M2-inv | 93.8±77.6 | -30.3 | 18 | 0.416 |
|  |  | M2-non-inv | 83.3±50.4 | -42.8 | 18 | 0.110 |
|  |  | M3-inv | 116.5±88.3 | -7.9 | 17.7 | 0.986 |
|  |  | M3-non-inv | 93.7±69.2 | -31.8 | 17.9 | 0.357 |
|  |  | M4-inv | 113.6±84.0 | -10.4 | 18.4 | 0.971 |
|  |  | M4-non-inv | 100.0±73.6 | -24.4 | 18.7 | 0.659 |
|  |  | M5-inv | 121.5±91.4 | -0.2 | 18.4 | 1 |
|  |  | M5-non-inv | 120.5±89.0 | -1.9 | 18.6 | 0.999 |
|  | **Semitendinosus RMS [% subMVC]** | M2-inv | 100.3±63.6 | 1.9 | 15.4 | 0.999 |
|  |  | M2-non-inv | 83.2±58.6 | -15.25 | 15.7 | 0.842 |
|  |  | M3-inv | 124.5±72.9 | 28 | 15.3 | 0.327 |
|  |  | M3-non-inv | 99.1±72.9 | 1.1 | 15.3 | 1 |
|  |  | M4-inv | 124.4±66.3 | 31.4 | 15.9 | 0.253 |
|  |  | M4-non-inv | 117.2±86.4 | 20.42 | 16 | 0.674 |
|  |  | M5-inv | 145.2±87.1 | 52.4 | 16.1 | 0.008** |
|  |  | M5-non-inv | 137.2±79.2 | 40.6 | 16.2 | 0.073 |
| **WEIGHT ACCEPTANCE** | **Vastus medialis RMS [% subMVC]** | M2-inv | 261.2±108.2 | -128.9 | 28.8 | 0.0001*** |
|  |  | M2-non-inv | 328.2±102.9 | -52.5 | 29.5 | 0.356 |
|  |  | M3-inv | 293.3±117.1 | -87.5 | 28.8 | 0.018* |
|  |  | M3-non-inv | 338.1±99.9 | -44.2 | 29 | 0.516 |
|  |  | M4-inv | 331.9±114.6 | -55.7 | 29.9 | 0.309 |
|  |  | M4-non-inv | 352.6±128.4 | -18 | 30.4 | 0.966 |
|  |  | M5-inv | 347.8±124.3 | -29.6 | 29.7 | 0.831 |
|  |  | M5-non-inv | 385.3±130.1 | 16.4 | 31.1 | 0.977 |
|  | **Vastus lateralis RMS [% subMVC]** | M2-inv | 222.7±109.6 | -144 | 31 | < 0.0001*** |
|  |  | M2-non-inv | 306.7±122.3 | -54.9 | 31.3 | 0.373 |
|  |  | M3-inv | 247.8±136.8 | -117.4 | 30.5 | 0.001*** |
|  |  | M3-non-inv | 283.9±125.5 | -83.9 | 30.8 | 0.045* |
|  |  | M4-inv | 244.0±110.6 | -125.1 | 31.8 | 0.0008*** |
|  |  | M4-non-inv | 358.0±118.2 | 2.9 | 31.7 | 0.999 |
|  |  | M5-inv | 275.8±139.3 | -98 | 31.8 | 0.016* |
|  |  | M5-non-inv | 321.2±145.1 | -47.7 | 33.3 | 0.563 |
|  | **Biceps femoris RMS [% subMVC]** | M2-inv | 97.4±60.3 | -7.5 | 14.3 | 0.977 |
|  |  | M2-non-inv | 107.6±64.1 | 3.1 | 14.2 | 0.998 |
|  |  | M3-inv | 106.9±70.7 | 2.5 | 14.1 | 0.999 |
|  |  | M3-non-inv | 104.6±56.6 | 0.1 | 14.2 | 1 |
|  |  | M4-inv | 98.8±62.1 | -5.9 | 14.6 | 0.990 |
|  |  | M4-non-inv | 102.3±64.8 | -2 | 14.9 | 0.999 |
|  |  | M5-inv | 104.2±77.1 | -0.3 | 14.6 | 1 |
|  |  | M5-non-inv | 110.0±66.1 | 6.8 | 14.7 | 0.985 |
|  | **Semitendinosus RMS [% subMVC]** | M2-inv | 97.2±68.0 | 42.9 | 11.3 | 0.001*** |
|  |  | M2-non-inv | 69.4±34.3 | 14.4 | 11.4 | 0.677 |
|  |  | M3-inv | 90.0±55.7 | 35 | 11.2 | 0.013** |
|  |  | M3-non-inv | 72.2±57.9 | 18 | 11.6 | 0.461 |
|  |  | M4-inv | 87.5±53.5 | 32.5 | 11.5 | 0.036* |
|  |  | M4-non-inv | 69.8±35.7 | 14.8 | 11.7 | 0.679 |
|  |  | M5-inv | 87.2±54.0 | 33.0 | 11.6 | 0.032* |
|  |  | M5-non-inv | 83.2±42.3 | 29.0 | 11.7 | 0.086 |
| **PUSH-OFF** | **Vastus medialis RMS [% subMVC]** | M2-inv | 317.9±143.1 | -111.4 | 38 | 0.025* |
|  |  | M2-non-inv | 462.6±124.8 | 36.8 | 39 | 0.856 |
|  |  | M3-inv | 309.2±173.9 | -94.9 | 38.4 | 0.088 |
|  |  | M3-non-inv | 429.2±161.3 | 12.4 | 38.3 | 0.995 |
|  |  | M4-inv | 386.8±154.0 | -47.7 | 38.6 | 0.700 |
|  |  | M4-non-inv | 432.5±137.5 | 15.6 | 39.9 | 0.991 |
|  |  | M5-inv | 369.7±158.1 | -64.2 | 40.7 | 0.481 |
|  |  | M5-non-inv | 469.3±149.8 | 51.5 | 41.4 | 0.693 |
|  | **Vastus lateralis RMS [% subMVC]** | M2-inv | 232.3±124.5 | -135.8 | 38 | 0.003** |
|  |  | M2-non-inv | 376.4±147.3 | 8.8 | 38.5 | 0.998 |
|  |  | M3-inv | 255.2±163.2 | -108.8 | 37.7 | 0.028* |
|  |  | M3-non-inv | 320.6±150.5 | -54.6 | 37.7 | 0.562 |
|  |  | M4-inv | 279.8±135.6 | -97.4 | 38.9 | 0.079 |
|  |  | M4-non-inv | 358.6±137.2 | -3.5 | 39 | 0.999 |
|  |  | M5-inv | 293.1±167.4 | -80.4 | 38.9 | 0.215 |
|  |  | M5-non-inv | 350.0±162.0 | -22.9 | 39.6 | 0.968 |
|  | **Biceps femoris RMS [% subMVC]** | M2-inv | 65.7±35.2 | -31.9 | 11.8 | 0.047* |
|  |  | M2-non-inv | 101.9±59.6 | 4.8 | 11.7 | 0.990 |
|  |  | M3-inv | 69.7±57.2 | -27.6 | 11.7 | 0.113 |
|  |  | M3-non-inv | 87.2±46.4 | -9.9 | 11.7 | 0.896 |
|  |  | M4-inv | 67.0±40.5 | -30.2 | 12 | 0.078 |
|  |  | M4-non-inv | 86.7±42.8 | -10.5 | 12.4 | 0.896 |
|  |  | M5-inv | 75.9±56.3 | -21.5 | 12 | 0.351 |
|  |  | M5-non-inv | 94.4±61.9 | -2.5 | 12.1 | 0.999 |
|  | **Semitendinosus RMS [% subMVC]** | M2-inv | 102.2±77.4 | 11.1 | 17 | 0.954 |
|  |  | M2-non-inv | 98.6±63.1 | 6.7 | 17.1 | 0.991 |
|  |  | M3-inv | 100.2±85.8 | 8.1 | 17 | 0.983 |
|  |  | M3-non-inv | 91.1±72.2 | -0.4 | 16.9 | 1 |
|  |  | M4-inv | 96.3±55.8 | 4.4 | 17.5 | 0.998 |
|  |  | M4-non-inv | 109.7±107.7 | 22.4 | 17.8 | 0.687 |
|  |  | M5-inv | 90.5±54.4 | -0.3 | 17.5 | 1 |
|  |  | M5-non-inv | 106.6±80.9 | 16.2 | 17.7 | 0.867 |

†ACL = anterior cruciate ligament; ‡SD = standard error; §SE = standard error; ¶M1-5 = measurement time point 1-5; ††inv = involved limb; ‡‡non-inv = non-involved limb; §§RMS = root mean square; ¶¶subMVC = submaximal voluntary contraction; Asterisks indicate statistical significance (* = *p* < 0.05, ** = *p* < 0.01, *** = *p* < 0.001)

**TABLE S5** Single-leg hop distance and limb symmetry index of the ACL group.

| ACL involved limb [cm] | ACL non-involved limb [cm] | Limb symmetry index |
| --- | --- | --- |
| 82.3 | 94.7 | 87 |
| 153.7 | 182.7 | 84 |
| 120.3 | 149.0 | 81 |
| 99.0 | 106.7 | 93 |
| 111.3 | 130.0 | 86 |
| 142.7 | 150.3 | 95 |
| 133.0 | 133.7 | 100 |
| 117.7 | 126.0 | 93 |
| 118.0 | 117.7 | 100 |
| 102.7 | 116.3 | 88 |
| 135.3 | 127.7 | 106 |
| 115.7 | 113.3 | 102 |
| 93.0 | 137.7 | 68 |
| 93.0 | 103.7 | 90 |
| 56.7 | 95.7 | 59 |
| 108.7 | 98.3 | 111 |
| 120.7 | 111.7 | 108 |
| 151.3 | 153.3 | 99 |
| 171.7 | 193.3 | 89 |
| 123.0 | 132.0 | 93 |
| 114.3 | 137.7 | 83 |
| 120.7 | 130.0 | 93 |
| 74.7 | 88.7 | 84 |
| 73.3 | 68.7 | 107 |
| 66.7 | 89.3 | 75 |
